# Hemodynamic instability caused by pneumorrachis and pneumomediastinum following epidural analgesia: a case report

**DOI:** 10.1186/s13256-024-04588-y

**Published:** 2024-05-29

**Authors:** Maria Fountoulaki, Emmanouil I. Kapetanakis, Niki Kouna, Nikolaos Papagiannis, Tatiana Sidiropoulou

**Affiliations:** 1 Second Department of Anesthesiology, “Attikon” University Hospital, National and Kapodistrian University of Athens, 1 Rimini Str., 12461 Athens, Greece; 2 Department of Thoracic Surgery, “Attikon” University Hospital, National and Kapodistrian University of Athens, 1 Rimini Str., 12461 Athens, Greece; 3 Second Department of Radiology, “Attikon” University Hospital, National and Kapodistrian University of Athens, 1 Rimini Str., 12461 Athens, Greece

**Keywords:** Anaesthesia, Epidural analgesia, Pneumomediastinum, Pneumorrachis, Hemodynamic instability

## Abstract

**Background:**

Pneumomediastinum and pneumorrachis are rare complications following epidural analgesia, that can either be asymptomatic or rarely can produce mild to moderate severity symptoms. Most reported cases regarding the presentation of these two entities with epidural analgesia concern asymptomatic patients, however there are cases reporting post-dural puncture headache and respiratory manifestations.

**Case presentation:**

We present a case where a combined lumbar epidural and spinal anesthesia was performed using the loss of resistance to air technique (LOR), on a 78-year-old Greek (Caucasian) male undergoing a total hip replacement. Despite being hemodynamically stable throughout the operation, two hours following epidural analgesia the patient manifested a sudden drop in blood pressure and heart rate that required the administration of adrenaline to counter. Pneumomediastinum, pneumorrachis and paravertebral soft tissue emphysema were demonstrated in a Computed Tomography scan. We believe that injected air from the epidural space and surrounding tissues slowly moved towards the mediastinum, stimulating the para-aortic ganglia causing parasympathetic stimulation and therefore hypotension and bradycardia.

**Conclusion:**

Anesthesiologists should be aware that epidural analgesia using the LOR to technique injecting air could produce a pneumomediastinum and pneumorrachis, which in turn could produce hemodynamic instability via parasympathetic stimulation.

## Background

Although epidural anesthesia and analgesia is considered a routine procedure it is not devoid of challenges and complications [1]. Proper placement of the epidural needle into the epidural space is paramount for success. The most common method to identify the epidural space is the loss of resistance (LOR) technique. Generally air or saline is used as a testing injected medium, however the former is associated with more adverse events, including incomplete analgesia, accidental dural puncture, postdural puncture headache, venous air embolism, subcutaneous emphysema and spinal cord compression [2]. Other complication such as pneumorrachis, pneumocephalus or pneumomediastinum are more rare, and usually result from the presence of air inside a closed compartment [2, 3]. However, it is still a widely utilized technique within the anesthetic community.

We report a case of iatrogenic pneumorrachis and accompanying pneumomediastinum producing profound hemodynamic instability, following spinal analgesia, and present an overview of relevant literature.

## Case presentation

A 78-year-old Greek (Caucasian) male, a smoker with a BMI of 25.7 kg/m^2^ who was classified as an American Society of Anesthesiologists (ASA) physical status grade III patient and therefore had a medium risk of anesthesia, was diagnosed with osteoarthritis of the left hip and thus was scheduled for an elective total hip replacement. His cardiac history included hypertension and dyslipidemia, with a normal pre-operative echocardiography examination (ejection fraction: 60%, with no abnormalities) and no history of pulmonary disease. He was also a type II diabetic, managed by oral medication (metformin) and had undergone a total knee replacement in the past without complications. His preoperative laboratory examination was within normal parameters and he was hemodynamically stable (pre-anesthesia assessment vitals: blood pressure: 137/67 mmHg, heart rate: 77 bpm, SpO2: 99%). The anesthesia plan included the use of a combined lumbar epidural and spinal anesthesia and analgesia.

Upon arrival in the operating room (OR) standard monitoring including continuous electrocardiography (ECG), oxygen saturation (SpO2) and non-invasive blood pressure measuring was commenced. An oxygen mask administering 6 lt/min was used throughout the procedure. The neuraxial spinal technique was performed aseptically, by a second year anesthesia trainee who had previously performed more than 40 procedures, supervised by a senior consultant anesthetist, with the patient in a sitting position at the level of the L4-L5 intervertebral space using a midline approach. After local anesthesia with 5 ml of 2% lidocaine, an 18-Gauge Tuohy needle attached to a syringe filled with approximately 8 ml of air was used. Intermittent pressure was applied to locate the epidural space using the loss of resistance to air technique.

The epidural space was located relatively easily after a single redirection of the needle. A needle-through-needle approach with an adjustable locking mechanism was used to locate the subarachnoid space. Cerebrospinal fluid (CSF) flowed freely through the spinal needle without the presence of blood. A mixture of 1,9 ml of ropivacaine 0.75% wit 0,2 ml (10 mcg) of fentanyl for a total volume of 2,1 ml was introduced into the subarachnoid space. The spinal needle was carefully removed, and the epidural catheter (Portex Epidural Minipack System 1, Smith’s Medical) was then placed in the epidural space. The spinal block was confirmed using the temperature differentiation method and both sensory and motor block were present. An arterial continuous blood pressure monitoring catheter was inserted in the patient’s radial artery prior to the commencement of the procedure. The patient exhibited stable baseline hemodynamics with a mean arterial pressure of 90 mmHg and a heart rate of 70–80 beats/min.

For surgery the patient was subsequently placed on the right lateral side with his left hip facing upwards. He received no intravenous anesthetic or analgesic medication throughout the procedure. Approximately 2 h after the spinal blockand and the initiation of the procedure the patients’ blood pressure suddenly dropped from 163/84 mmHg to 82/45mmHg and his heart rate decreased to 45 bpm. He was alert and responsive but stated that he felt dizziness. Sinus bradycardia was apparent on the ECG. The anesthesiologist in charge administered promptly 5 mg of ephedrine and a total of 1 mg of phenylephrine in consecutive boluses over a period of 30 s. Simultaneously O2 administration via a face mask was increased to 15 lt/min and intravenous fluid administration was increased. There was no improvement in the patient’s hemodynamic state and his blood pressure dropped further to 55/35 mmHg and the patient then became unresponsive. Fearing imminent patient arrest, the anesthetic incident alert was raised for additional help and 1mg of adrenaline was given intravenously by the consultant anesthetist. This produced an immediate rise in the blood pressure (up to a peak of 183/88 mmHg which required the administration of small 0.05 mg/ml boluses of nitroglycerin to prevent extreme hypertension) and the patient’s mental status promptly returned to normal. An immediate cardiology assessment was requested. A point of care echocardiogram demonstrated no new cardiac abnormalities or changes compared to the patients’ preoperative assessment echo. During the episode, there was a small drop in the patient’s SpO2 from 97% from 100% but his arterial blood gasses were normal. No dyspnea or chest pain was recorded during or after the episode.

**Fig. 1:**
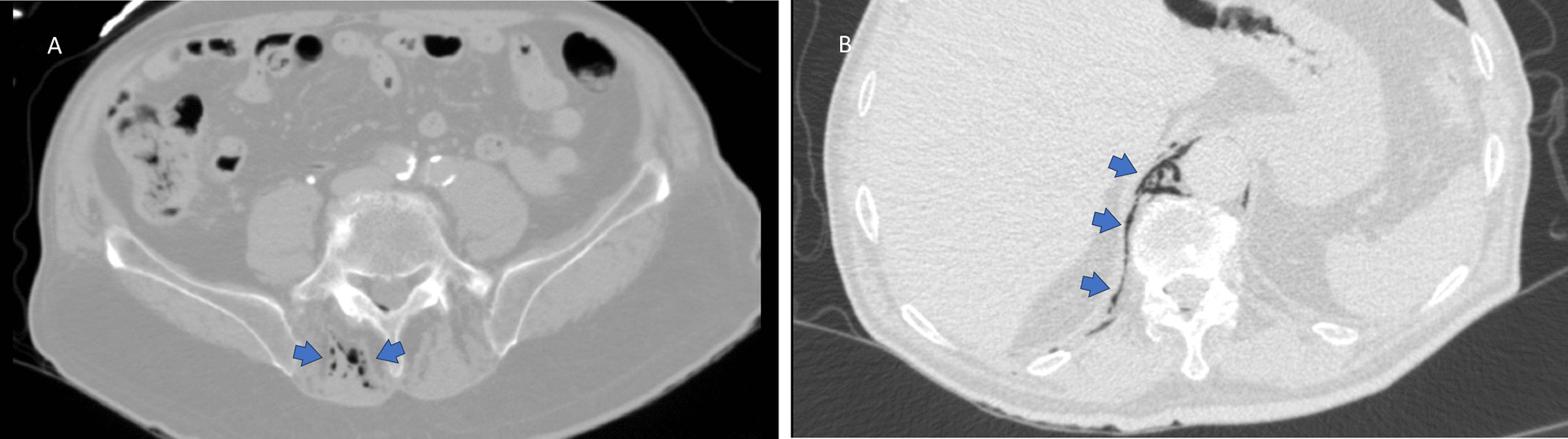
CT Scan of the abdomen **A** Free air (arrows) present in the subcutaneous tissue at the puncture site and in the epidural space; **B** Air (arrows) present in the paravertebral soft tissue and around the abdominal aorta

**Fig. 2:**
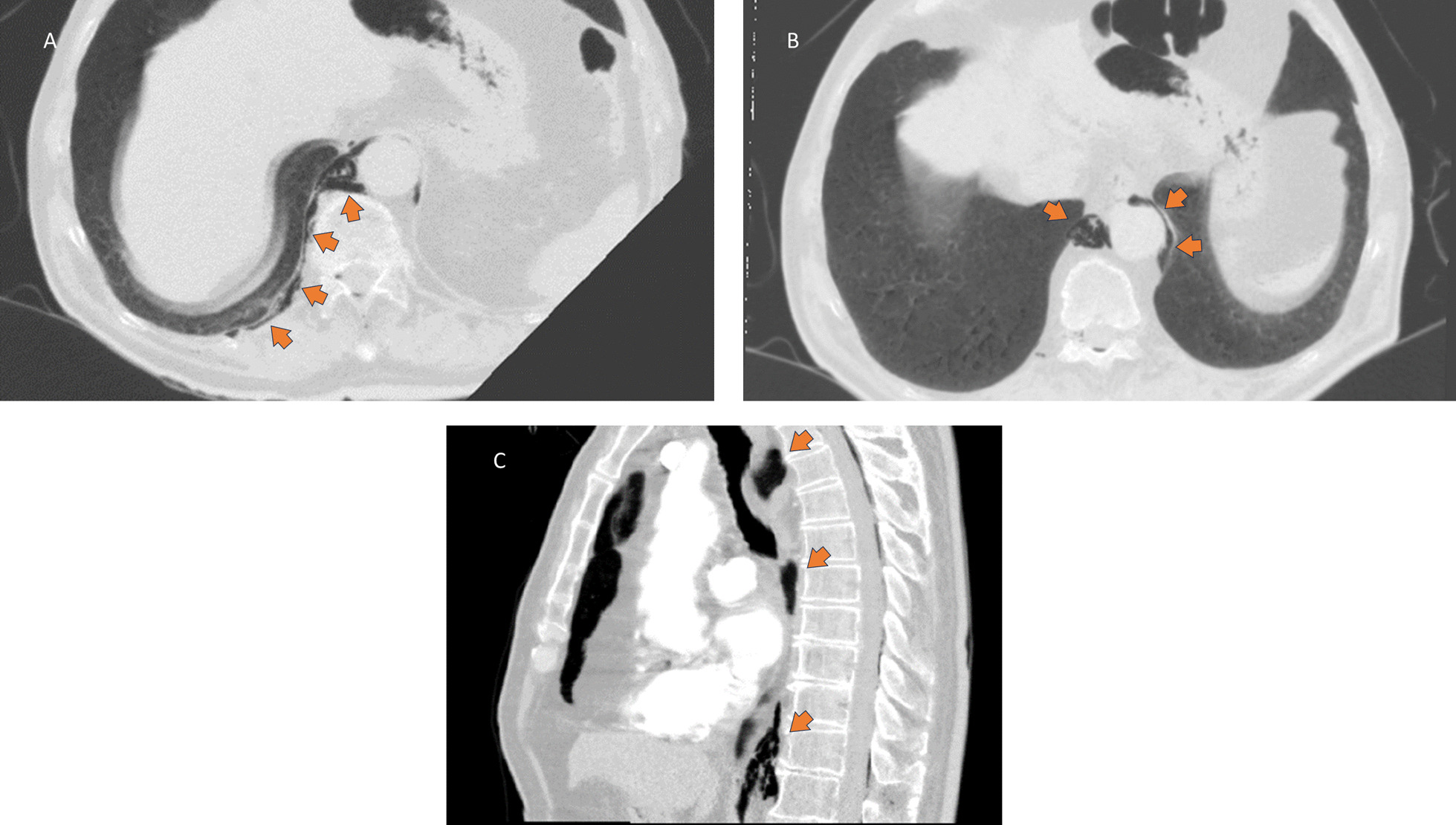
CT Scan of the abdomen/chest **A** Free air (arrows) in the paravertebral subcutaneous tissue and around the aorta (demonstration of tissue continuation); **B** Free air (arrows) present in the mediastinum (pneumomediastinum) surrounding the aorta; **C** Sagittal view (arrows show air in different levels)

After the end of the procedure, the patient was admitted to the post-anesthetic care unit (PACU), where continuous monitoring of his vital signs was performed. No additional epidural medication was administered and his epidural catheter was removed. A computed tomography pulmonary arteriogram (CTPA) was performed, which was negative for a pulmonary embolism. A chest and abdomen CT was also performed, which demonstrated air in the subcutaneous tissue, the intermuscular space, the spinal canal around the spinal cord (pneumorrachis), at the left side of the L4 and L5 vertebral bodies and inside the mediastinum para-aortically (Figs. 1 and 2).

Post operatively, the patient underwent a complete and thorough cardiovascular assessment including a clinical examination by a cardiologist, cardiac echocardiography, cardiac enzymes and a carotid triplex ultrasound with no abnormal findings. He was put on bed rest under continuous cardiac monitoring (blood pressure, heart rate and ECG) with no other such episodes of hemodynamic instability or hypotension been recorded. He was discharged home on the fifteenth postoperative day and was followed up as per routine clinical practice by his orthopedic surgeon.

## Discussion

Pneumorrachis and pneumomediastinum are medical conditions characterized by the abnormal presence of air in a closed compartments. Pneumorrachis refers to the presence of air in the subarachnoid or the epidural space, which can occur as a result of trauma, infection, or medical procedures. This condition is often associated with vertebral fractures, spinal surgery or iatrogenic associated with the epidural LOR to air technique. However, one should bear in mind that the epidural space has no fascial barriers separating it from other structures, therefore air can freely migrate from the intervertebral foramina towards the posterior mediastinum causing pneumomediastinum or pneumothorax, arising to complications similar to the one presented here. Pneumomediastinum can most often develop from various other causes, including trauma, underlying lung diseases, or spontaneously due to alveolar rupture. Both pneumorrachis and pneumomediastinum are typically diagnosed through imaging studies, specifically CT scan, and may require medical intervention depending on the underlying cause and severity of symptoms. Management may involve addressing the root cause, relieving pressure, and providing supportive care to the affected individuals. However pneumorrhachis is usually asymptomatic and reabsorbs spontaneously [4–6]. Therefore, patients with pneumorrhachis are commonly managed conservatively. Pneumomediastinum can also be self-resolving but can also be associated with serious morbidity and mortality [7, 8].

In this reported case, it is suspected that the air used in the epidural technique was slowly introduced into the mediastinum (pneumomediastinum) via the continuation of the peri-muscular tissue plains. The pressurized air, accumulated in the paraspinal muscles and in the spinal canal, dissected along the fascial planes into the mediastinum. Even that small amount of air triggered the parasympathetic ganglia around the aorta and thus, caused parasympathetic stimulation and therefore hypotension and sinus bradycardia that required the administration of adrenaline to be reversed. We need to note that at the time the hemodynamic instability occurred intra-operatively the anesthetic team was unaware of this mechanism which was only subsequently revealed following additional imaging and investigation. Therefore at the time standard anesthetic protocols for the management of hypotension were followed [9]. As mentioned, as per protocol, initially fluid administration was increased and ephedrine and phenylephrine were administered up to maximal recommended dosages [9]. However the hemodynamic instability did not respond to these measures and therefore since all other vassopressors had failed and fearing an ensuing cardiac arrest, adrenaline was administered by the consultant anesthetist. Adrenaline is both an alpha and beta-adrenergic agonist, and although it may seem as a conventional drug, it remains “the treatment of choice in cases of anaphylaxis during anesthesia” [9]. Given the imminent complete hemodynamic collapse of the patient its administration was crucial in restoring the hemodynamic parameters.

Similarly to our case, Shaik *et al.* reported pneumorrhachis and pneumothorax in a 26-year old primi gravida which was scheduled for a cesarean section [10]. An epidural was performed prior to the procedure, again using the LOR with air technique. After an uneventful cesarean section and delivery, the patient developed tachypnea and low oxygen saturation in the post-anesthesia care unit. She was however hemodynamically stable, and was managed conservatively having a full recovery. In another instance, Lim *et al.* reported pneumomediastinum and pneumorrhachis at the C2-T10 levels and paravertebral muscle emphysema in a 56-year old male following a thoracic epidural block using the LOR to air technique [11]. This patient however was asymptomatic and was managed conservatively recovering fully. Naik *et al.* reported asymptomatic pneumorrhachis and pneumomediastinum in the thoracic region in a 57-year-old male scheduled for distal gastrectomy. A thoracic epidural was also performed for post-operative analgesia. The patient was managed conservatively and he too recovered fully [12].

In our case the patient also recovered fully and luckily did not suffer any lasting sequelae. Post the event he underwent a complete cardiovascular assessment which demonstrated he did not suffer any long-term implications.

## Conclusion

In conclusion, the possibility of pneumomediastinum and pneumorrhachis is a possible, although rare complication, after an epidural block, especially with the LOR with air technique. Cautious performance of the block, minimizing the air injected in the epidural space, as well as timely recognition and treatment of the symptoms and considering replacing the use of air with saline are important for the prevention and management of this possible complication and have been suggested in the literature [13].

## Data Availability

All relevant information regarding this case are included in this article. However, any additional information that may be needed are available from the corresponding author upon reasonable request.

## Abbreviations

LOR: Loss of resistance
ASA: American Society of Anesthesiologists
OR: Operating room
ECG: Electrocardiography
CCSF: Cerebrospinal fluid
PACU: Post-anesthetic care unit
CTPA: Computed tomography pulmonary arteriogram
